# Are Red Blood Cell Distribution Width and Platelet Count Useful for Diagnosing Periprosthetic Joint Infection in Patients Undergoing Re‐Revision Arthroplasty

**DOI:** 10.1111/os.14219

**Published:** 2024-10-01

**Authors:** Yangming Zhang, Qiyu Xie, Boyi Jiang, Wenyu Jiang, Hong Xu, Zongke Zhou

**Affiliations:** ^1^ Department of Orthopedics and Orthopedic Research Institute, West China Hospital Sichuan University Chengdu People's Republic of China; ^2^ West China School of Medicine Sichuan University Chengdu People's Republic of China

**Keywords:** Diagnosis, Periprosthetic Joint Infection, Platelet, Red Blood Cell Distribution Width, Re‐revision Arthroplasty

## Abstract

**Objective:**

Accurate and prompt identification of periprosthetic joint infections (PJIs) is critical prior to re‐revision arthroplasty to ensure optimal surgical outcomes. Among routinely measured blood indices, red blood cell distribution width (RDW) and platelet count (PLT) have shown strong correlations with infection presence. This study aimed to assess the utility of RDW and PLT for diagnosing PJI in patients scheduled for re‐revision arthroplasty.

**Methods:**

This retrospective research encompassed all patients who underwent re‐revision hip or knee arthroplasty at our institution from 2008 to 2022. Participants were categorized into either the PJI (*n* = 41) or the non‐PJI (*n* = 47) group following the guidelines established in the 2013 International Consensus Meeting on PJI. In this analysis, RDW and PLT counts were evaluated alongside conventional inflammatory markers, including C‐reactive protein (CRP) and erythrocyte sedimentation rate (ESR). The efficacy of these diagnostics was evaluated by the area under the receiver operating characteristic (ROC) curve ([area under the curve AUC]).

**Results:**

RDW demonstrated a modest AUC of 0.678 with sensitivity at 61.0% and specificity at 71.7%, using a threshold of 14.5%. PLT was on par with ESR, showing an AUC of 0.773, and both sensitivity and specificity around 73% at a threshold of 201 × 10^9^/L. CRP presented the highest diagnostic accuracy with an AUC of 0.815, achieving a sensitivity of 82.9% and specificity of 73.9% at a 6.9 mg/L threshold, surpassing ESR's AUC of 0.754. None of the biomarkers, individually or combined, outperformed CRP alone (*p* > 0.05).

**Conclusions:**

In the context of re‐revision arthroplasty, RDW and PLT demonstrate limited efficacy as diagnostic biomarkers for PJI. However, CRP retains its reliability as a biomarker when the diagnostic threshold is appropriately recalibrated.

## Graphical abstract

Receiver operating characteristic curves. (A) C‐reactive protein (CRP), erythrocyte sedimentation rate (ESR), red blood cell distribution width (RDW), platelet count (PLT) on their own. (B) Combinations of two biomarkers. (C) Combinations of three biomarkers. (D) Combination of four biomarkers.

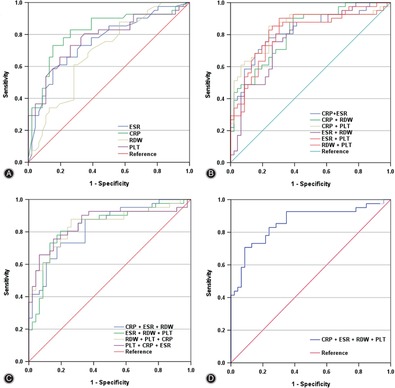

## Introduction

Periprosthetic joint infection (PJI) is the most formidable complication following total knee and hip arthroplasties, often resulting in extended hospitalizations, increased medical costs, and potential long‐term disabilities.^1^ By 2030, it is anticipated that there will be around 268,200 knee and 97,700 hip revision surgeries required in the United States.^2^ Managing PJI after these revision surgeries becomes particularly complex due to bone deterioration and tissue scarring, which may compromise the outcomes and strain the relationship between patients and healthcare providers.^3^ Early and precise identification of PJI prior to re‐revision surgeries is essential for choosing the right treatment strategies and managing patient expectations effectively.^4^

In the realm of diagnostics, synovial fluid analysis is indispensable for confirming PJI, but blood‐based biomarkers offer a practical, rapid, and less invasive alternative.^5^ Current international guidelines for PJI diagnosis advocate for the use of erythrocyte sedimentation rate (ESR), serum C‐reactive protein (CRP), and D‐dimer.^6^ Nonetheless, these markers might not show significant elevation in cases of infection with low‐virulence organisms. Moreover, the diagnostic effectiveness of D‐dimer in PJI scenarios is still a matter of debate.^7^ This has spurred interest in developing new, rapid, and reliable hematologic biomarkers to improve diagnostic accuracy for PJI.

Exploration into red blood cell distribution width (RDW) and platelet count (PLT) as potential diagnostic tools is ongoing.^8^, ^9^ Inflammatory states such as acute pancreatitis or severe infections often lead to alterations in red blood cell morphology, which are captured by RDW—an indicator of red blood cell size variability. Research has shown that RDW can be indicative of infection risks and adverse outcomes post‐liver transplantation scenarios.^10^, ^11^ RDW is also capable of predicting the development of liver failure in HEV patients.^12^ Hence, RDW might also have predictive value in revision arthroplasty. However, the precise diagnostic value of RDW in PJI is yet to be fully explored. Platelets are anucleated blood cells derived from bone marrow megakaryocytes and the second most abundant cell in the blood after red blood cells. They are best known for their critical role in clotting, but they also play an important role in the body's response to inflammation and infection.^13^ Gasparyan *et al*. reported that the numbers and volume are highly associated with the inflammation level of rheumatoid arthritis.^14^ Ozcelik *et al*. reported that platelet count is an easily accessible and inexpensive biomarker for the diagnosis of COVID‐19.^15^ Hence, platelets may be useful in diagnosing PJI, especially before primary revision surgeries, although their role prior to re‐revision surgeries remains to be determined.^16^, ^17^ This study is aimed at (i) identifying the value of RDW and PLT for diagnosing PJI compared with traditional inflammatory markers, namely, CRP and ESR; and (ii) evaluating the value of RDW and PLT combined with CRP and ESR for diagnosing PJI.

## Methods

### Study Design

The Institutional Review Board (IRB) provided ethical clearance for this retrospective investigation, conducted at a single institution (IRB Approval No. 2020‐1004). Due to the study's retrospective nature and the absence of any adverse effects on participant health, the IRB granted a waiver for the informed consent requirement. Throughout the study and prior to data analysis, all patient identifiers were anonymized. This trial was registered with the Chinese Clinical Trial Registry under the identifier ChiCTR2000039989.

### Patients

We identified patients at our institute who underwent re‐revision hip or knee arthroplasty from 2008 to December 2022 because of either aseptic failure or PJI following revision arthroplasty. The exclusion criteria include (i) patients who had re‐implantation surgery, as this operation is part of the two‐stage revision process used to treat PJI, and the source of infection may be ambiguous, suggesting either a new infection or a recurrence^18^; (ii) patients who had re‐revision arthroplasty due to periprosthetic fracture or dislocation so as to minimize the influence of trauma on inflammatory markers^19^, ^20^; and (iii) patients who were followed up for less than 1 year.

### Diagnostic Definition of Periprosthetic Joint Infection

For our study, patients were categorized into either the PJI group or the non‐PJI group, adhering to the criteria set forth in the 2013 International Consensus Meeting (ICM) on PJI rather than the 2018 guidelines. This decision was due to the absence of synovial fluid α‐defensin testing capabilities at our institution and the ongoing debate regarding the reliability of serum D‐dimer levels in diagnosing PJI.^5^, ^21^ Furthermore, we exclusively considered noninfected patients who had been monitored for a minimum duration of 1 year, to reduce the likelihood of overlooking cases with infections.

### Laboratory Tests

Upon each patient's initial admission, routine examinations such as CRP, ESR, and complete blood counts, which include RDW and PLT measures, were administered. Individuals suspected of PJI based on factors including prior PJI‐related surgeries, joint symptoms, and notably elevated CRP and ESR levels (exceedingly twice the normal upper limit), along with clinical and radiographic evidence of joint involvement, were selected for further diagnostic procedures. Aspiration of joints was a key diagnostic step. Technicians specializing in ultrasound from the ultrasound department performed hip aspirations using Doppler guidance, while surgeons generally handled knee aspirations in a dedicated room. Stringent sterilization techniques were employed during all aspiration procedures to avoid introducing new infectious agents into the joint.

All aspirated samples were immediately transported to the Department of Laboratory Medicine of our institution for prompt processing. The tests conducted on these samples included aerobic and anaerobic cultures, as well as analysis of joint fluid for white blood cell count, differential neutrophil count, and percentage of polymorphonuclear neutrophils. Should the aspirate volume be adequate, both types of cultures and full joint fluid analysis were performed; if not, only the cultures were carried out. Moreover, during any subsequent surgical revisions, at least four soft tissue samples from around the implant area were taken for both histological examination and culture, with neutrophil assessments also conducted. An infection was suggested if there was a presence of more than five neutrophils per high‐power field across five fields.

### Outcomes

Pre‐revision serum CRP, ESR, RDW, and PLT levels, as well as the ultimate diagnosis of either aseptic failure or PJI for re‐revision arthroplasty, were among the outcomes assessed in this investigation. The demographic information that was recorded included age and sex as well as pertinent comorbidities like diabetes, hypertension, psoriasis, chronic obstructive pulmonary disease, and coronary heart disease. Inflammatory diseases such as psoriasis, rheumatoid arthritis, and ankylosing spondylitis were also examined for any confounding factors,^18^ as was the interval between the first and the second re‐revision arthroplasties.

### Sample Size Estimation

We calculated the minimal sample size using MedCalc 12.7 (MedCalc Software Ltd., Ostend, Belgium). For the detection of PJI, the following AUCs were observed: CRP, 0.887; ESR, 0.842; PLT, 0.746; RDW did not have an associated AUC value. Since no sufficient information about the AUC was found in relation to RDW, the calculation was carried out on the AUC of PLT of 0.746 at a magnitude of type I error of 0.05 and a type II error of 0.1. As a result, it was required to study each group with at least 26 participants.

### Statistical Analyses

Means and standard deviations (SDs) were used to summarize normally distributed continuous variables, while differences between the two groups were assessed using Student's *t*‐test. For continuous variables with non‐normal distributions or when the assumption of variance homogeneity was not met, the Wilcoxon Mann–Whitney *U* test was employed, and the median along with interquartile ranges (IQR) was utilized as the relevant statistical measures. Proportions and percentages for categorical data were reported and compared between two groups using Pearson's chi‐squared test or Fisher's exact test when applicable. The significance threshold for each test was set at less than 0.05. Receiver operating characteristic curves were applied to delineate the sensitivity and specificity relationship of the biomarkers under investigation. Optimal cutoff values of tested biomarkers were obtained using the Youden index. Comparisons of the area under the curves (AUCs) of these biomarkers, whether combined or individually, to the standard benchmark AUC for CRP, established by the provided MedCalc version 12.7, were conducted using a *z*‐test. Additionally, the 2013 ICM guidelines for PJI were adhered to in assessing the clinical utility of CRP and ESR,^19^ with thresholds established at 10 mg/L and 30 mm/h, respectively. Calculations for positive and negative predictive values were also performed. All these analyses were conducted with the Statistical Package for the Social Sciences (SPSS) version 24 (IBM, Armonk, NY, USA).

## Results

### Baseline Characteristics

**FIGURE 1:**
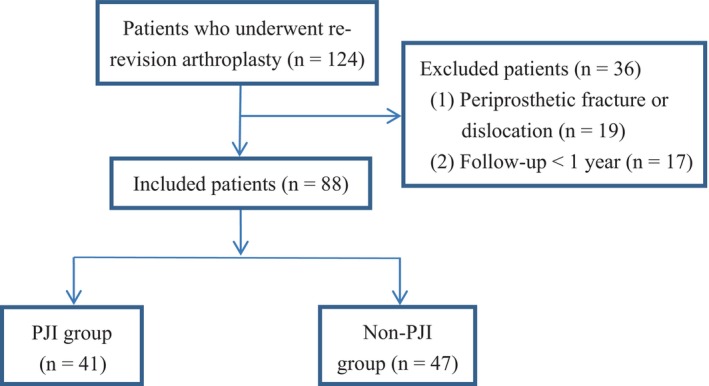
Flowchart of patient enrollment.

In this study, initially 124 participants were registered. Of these, 36 were subsequently removed from the study due to 19 suffering from periprosthetic fractures or dislocations and 17 having follow‐up durations shorter than 1 year. This left 88 participants for inclusion in the final analysis: 41 were diagnosed with PJI, and 47 experienced aseptic failures (Figure 1). Comparative analysis of demographic and comorbidity profiles between the PJI and non‐PJI cohorts showed no notable differences. However, a significant difference was evident in the proportion of hip joint infections, with the PJI cohort showing a lower incidence (68.3%) than the non‐PJI group (93.6%) (*p* = 0.002). The interval time between the first and the second re‐revision of the PJI group was significantly shorter than that of the non‐PJI group (*p* = 0.005) (Table 1). Within the PJI subset, 27 patients had confirmed infections (65.9%), predominantly caused by *Staphylococcus aureus*.

**TABLE 1:** Characteristics of the infected and noninfected groups.

Variables	PJI group (*n* = 41)	Non‐PJI group (*n* = 47)	*t* or *χ* ^2^ value	*p* value
Demographic characteristics				
Age, year	68.8 ± 13.6	61.5 ± 11.2	−0.852	0.397
Female	21 (51.2)	24 (51.1)	<0.001	0.988
Comorbidities				
Hypertension	7 (17.1)	12 (25.5)	0.925	0.336
Diabetes	5 (12.2)	6 (12.8)	0.007	0.936
COPD	6 (14.6)	1 (3.7)	3.126	0.077
CHD	1 (2.4)	2 (4.3)	<0.001	1.000
Inflammatory diseases	6 (14.6)	4 (8.6)	0.321	0.571
Involved joint (hip)	28 (68.3)	44 (93.6)	9.440	0.002*
The interval between the first and the second re‐revision (month)	111.62 ± 87.36	162.69 ± 77.43	2.908	0.005*

*Note*: Values are *n* (%) or mean ± SD, unless otherwise noted.

Abbreviations: CHD, coronary heart disease; COPD, chronic obstructive pulmonary disease; SLE, systemic lupus erythematosus.

* *p <* 0.05.

### Values of Tested Biomarkers

Subsequent evaluations focused on comparing four specific biomarkers between the two groups. Our results revealed that the PJI cohort had significantly elevated levels of CRP [21.2 (9.8–56.4) mg/L *vs* 3.7 (2.4–7.6) mg/L; *p* < 0.001], ESR [52.0 (29.5–78.5) mm/h *vs* 12.0 (4.8–24.0) mm/h; *p* < 0.001], RDW [(14.7 ± 1.1)% *vs* (13.9 ± 1.2)%; *p* = 0.002], and PLT [(254.6 ± 91.9) *vs* (173.5 ± 55.8) 10^9/L; *p* < 0.001] compared with the non‐PJI group (Table 2).

**TABLE 2:** Tested markers in the infected and noninfected groups.

Potential markers	PJI group (*n* = 41)	Non‐PJI group (*n* = 47)	*t* or χ^2^ value	*p* value
CRP (mg/L)	21.2 (9.8–56.4)	3.7 (2.4–7.6)	5.217	<0.001*
ESR (mm/h)	52.0 (29.5–78.5)	12.0 (4.8–24.0)	4.404	<0.001*
RDW (%)	14.7 ± 1.1	13.9 ± 1.2	3.241	0.002
PLT (×10^9^/L)	254.6 ± 91.9	173.5 ± 55.8	5.075	<0.001

*Note*: Data were presented as median (IQR) or mean ± SD.

Abbreviations: CRP, C‐reactive protein; ESR, erythrocyte sedimentation rate; IQR, interquartile range; PLT, platelet count; RDW, red blood cell distribution width.

* *p* < 0.05.

### Diagnostic Value of Tested Markers Individually

**FIGURE 2:**
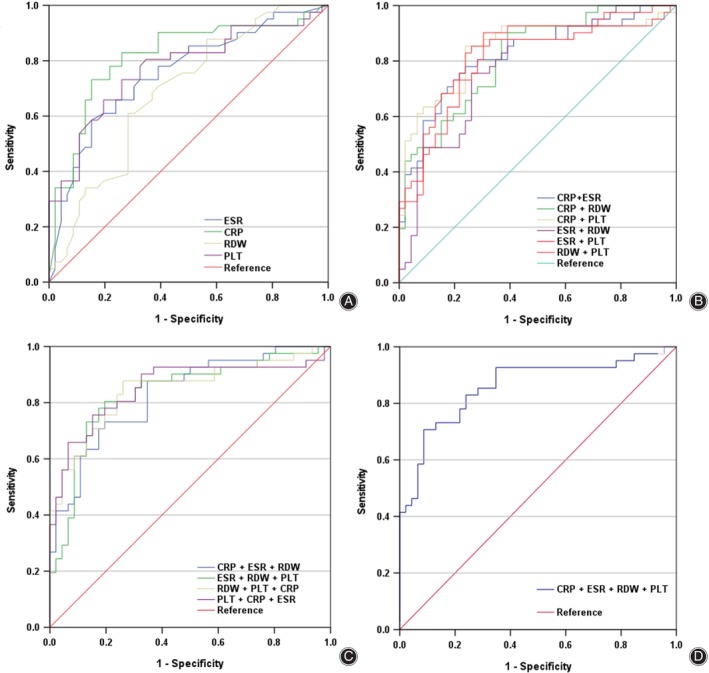
Receiver operating characteristic curves. (A) C‐reactive protein (CRP), erythrocyte sedimentation rate (ESR), red blood cell distribution width (RDW), and platelet count (PLT) on their own. (B) Combinations of two markers. (C) Combinations of three markers. (D) Combination of four markers.

The diagnostic performance of these biomarkers in detecting PJI in patients slated for re‐revision hip or knee arthroplasty was further assessed through additional analyses (Table 3; Figure 2A). Among the biomarkers, serum CRP exhibited the highest AUC of 0.815 (95% CI 0.720–0.910) and was able to differentiate PJI with an acceptable sensitivity of 73.2% and specificity of 73.9%, as determined by the established cutoff value of 10 mg/L.^19^ The sensitivity improved to 82.9% using the optimal cutoff of 6.9 mg/L determined by the Youden index for this study. ESR differentiated PJI with an AUC of 0.754 (95% CI 0.651–0.875) but did not differ significantly from CRP (*p* = 0.236). ESR demonstrated a sensitivity within an acceptable range of 73.2% but exhibited a lower specificity of 60.9% at the prescribed cutoff of 30 mm/h.^19^ Nevertheless, the sensitivity decreased to 58.5% at a specificity of 84.8%, using the optimal cutoff value of 45.5 mm/h, as established by the Youden index. RDW showed limited effectiveness in distinguishing PJI, indicated by an AUC of 0.678 (95% CI 0.576–0.798), with a sensitivity of 61.0% and a specificity of 71.7% at the established cutoff of 14.5%. In contrast, PLT effectively discriminated PJI, as evidenced by an AUC of 0.773 (95% CI 0.671–0.874), with sensitivities of 73.2% and specificities of 73.9% at the recommended cutoff of 201 × 10^9^/L.

**TABLE 3:** Diagnostic performance of the tested markers individually.

Potential markers	AUC (95% CI)	Youden index	Predictive cutoff	Sensitivity (%)	Specificity (%)	PPV (%)	NPV (%)	*Z* value	*p* value compared with CRP
CRP (mg/L)	0.815 (0.720–0.910)	0.515	10.0^a^	73.2	73.9	71.0	76.0	–	–
		0.568	6.9^b^	82.9	73.9	73.5	83.2		
ESR (mm/h)	0.754 (0.651–0.857)	0.341	30.0^a^	73.2	60.9	62.0	72.3	1.117	0.264
		0.433	45.5^b^	58.5	84.8	77.1	70.1		
RDW (%)	0.678 (0.576–0.798)	0.327	14.5^b^	61.0	71.7	65.3	67.8	1.799	0.072
PLT (×10^9^/L)	0.773 (0.671–0.874)	0.471	201^b^	73.2	73.9	70.1	76.0	0.687	0.492

Abbreviations: 95% CI, 95% confidence interval; AUC, area under the receiver operating characteristic curve; CRP, C‐reactive protein; ESR, erythrocyte sedimentation rate; NPV, negative predictive value; PLT, platelet count; PPV, positive predictive value; RDW, red blood cell distribution width.

^a^ Predictive cutoffs determined based on the Musculoskeletal Infection Society criteria (2013).

^b^ Predictive cutoffs determined based on the Youden index.

### Diagnostic Value of Tested Markers Combined with Each Other

Ultimately, we evaluated the diagnostic utility of various combinations of CRP, ESR, RDW, and PLT for diagnosing PJI (Table 4; Figure 2B–D). Our findings suggest that the AUCs of the combinations of two, three, or four biomarkers, although subject to some variability, did not significantly deviate from that of CRP alone.

**TABLE 4:** Diagnostic performance of the tested markers in combination.

Combinations	AUC (95% CI)	Youden index	Sensitivity (%)	Specificity (%)	PPV (%)	NPV (%)	*Z* value	*p* value compared with CRP
Combination of two markers								
CRP + ESR	0.821 (0.731–0.911)	0.541	78.0	76.1	74.0	80.8	0.284	0.777
CRP + RDW	0.815 (0.728–0.903)	0.511	90.2	60.9	66.8	87.7	0.025	0.980
CRP + PLT	0.845 (0.756–0.933)	0.615	85.4	76.1	75.7	85.7	0.822	0.411
ESR + RDW	0.777 (0.679–0.875)	0.495	75.6	73.9	71.7	77.6	0.654	0.513
ESR + PLT	0.813 (0.781–0.907)	0.598	90.2	69.6	72.1	89.1	0.035	0.972
RWD + PLT	0.834 (0.733–0.914)	0.593	85.4	73.9	74.1	85.3	0.154	0.878
Combination of three markers								
CRP + ESR + RDW	0.830 (0.745–0.915)	0.536	73.2	80.4	76.5	77.5	0.553	0.580
ESR + RDW + PLT	0.834 (0.744–0.923)	0.609	80.5	80.4	78.2	82.5	0.365	0.715
RDW + PLT + CRP	0.846 (0.761–0.932)	0.617	87.8	73.9	95.8	47.0	0.817	0.414
PLT + CRP + ESR	0.853 (0.766–0.940)	0.604	75.6	84.8	81.3	79.9	1.099	0.272
Combination of four markers								
CRP + ESR + RDW + PLT	0.859 (0.777–0.941)	0.620	70.7	91.3	87.6	78.1	1.234	0.217

Abbreviations: 95% CI, 95% confidence interval; AUC, area under the curve; CRP, C‐reactive protein; ESR, erythrocyte sedimentation rate; NPV, negative predictive value; PLT, platelet count; PPV, positive predictive value; RDW, red blood cell distribution width.

## Discussion

### The Main Findings and Significance of the Study

To the best of our knowledge, this study is the inaugural exploration of the diagnostic capabilities of RDW and PLT in identifying PJI in patients undergoing re‐revision arthroplasty. Our findings indicate that both RDW and PLT are insufficient biomarkers for the diagnosis of PJI in this patient population. Conversely, serum CRP, utilizing a low cutoff, demonstrates efficacy in diagnosing PJI, showing satisfied sensitivity and adequate specificity among these patients.

### The Value of RDW for Diagnosing PJI and Its Roles in Inflammation and Infection

Revision arthroplasty serves as a crucial method to address failure following primary joint arthroplasty.^22^ However, it is susceptible to failure for several reasons, including aseptic loosening, periprosthetic fracture, recurrent dislocation, and PJI, the latter being the most common cause of failure that necessitates re‐revision arthroplasty. Repeated operations render re‐revision arthroplasty particularly challenging, underscoring the critical importance of accurately determining the presence of PJI prior to re‐revision arthroplasty for optimizing postoperative outcomes.

RDW values are routinely obtained as part of a complete blood cell count, offering the advantages of cost‐effectiveness, convenience, and minimal additional labor. Under physiological or pathological conditions, red blood cell volume can deviate from the normal range of 80–100 fl, reaching up to 150 fl or decreasing to 60 fl without membrane rupture.^23^ RDW quantifies the degree of heterogeneity in red blood cell volume, typically ranging between 11.5% and 14.5%.^24^ Elevated RDW levels are linked to oxidative stress and inflammation, noted in conditions such as Down syndrome, compromised lung function, and dialysis.^25^, ^26^ Numerous studies have associated elevated RDW levels with adverse outcomes across diverse clinical settings, including cardiovascular disease, peripheral artery disease, heart failure, atrial fibrillation, stroke, and increased mortality risk in hospitalized adults with SARS‐CoV‐2 infection,^27^ as well as postoperative infection in liver transplant recipients,^11^ and adverse outcomes following revision arthroplasty.^10^ However, our study findings suggest that RDW lacks reliability in identifying PJI in patients undergoing re‐revision arthroplasty. This may be attributed to the localized nature of PJI as a joint infection, resulting in a lesser degree of systemic inflammation and stress response. Additionally, RDW changes gradually, reflecting volume variance in a cell population that turns over at a rate of about 1%–2% per day,^28^ rendering it less responsive to acute changes during infections.

### The Value of PLT for Diagnosing PJI and Its Roles in Inflammation and Infection

PLT, derived from megakaryocytes, plays a crucial role in hemostasis and thrombosis.^28^ Notably, PLT also exhibits a close association with inflammation and infection.^13^ PLT, rich in pro‐inflammatory agents and capable of releasing highly active microparticles, is pivotal in antigen presentation and combating infection.^29^ Our previous research revealed a sensitivity of 57.5% and high specificity of 83.1% for PLT,^12^ while the current study demonstrates a comparable AUC for PLT with ESR, exhibiting a satisfactory sensitivity of 73.2% and specificity of 73.9%. However, as a primary screening biomarker for detecting PJI, CRP provides higher sensitivity (82.9%) and satisfactory specificity (73.9%), thus making it more reliable.

### Limitations and Strengths of this Study

Several limitations of our study should be considered when interpreting our results. First, it was a single‐center retrospective study, and some data were missing for certain individuals. Nevertheless, our cohort data offered sufficient evidence to determine that RDW and PLT are inadequate for diagnosing PJI in patients prior to re‐revision arthroplasty. Second, the sample size in our study was limited, necessitating validation and expansion of our findings through studies with larger sample sizes. Third, the small sample size precluded further assessment of the impact of comorbidities such as rheumatoid arthritis and ankylosing spondylitis on the diagnostic utility of RDW and PLT.

## Conclusions

While RDW and PLT levels are higher in patients undergoing re‐revision arthroplasty for PJI than in those with aseptic mechanical failure, both biomarkers, whether applied singly or in combination, show limited reliability in diagnosing PJI. Serum CRP, using a low cutoff, proves effective in these patients.

## Author Contributions

Yangming M. Zhang and Qiyu Y. Xie applied for ethical clearance and registered this study, drafted the work, and revised it critically for important intellectual content. Boyi Y. Jiang and Wenyu Y. Jiang collected data, analyzed, and interpreted data for the work. Hong Xu and Zongke K. Zhou contributed to the conception and design of the work, participated in the final approval of the version to be published, and revised the manuscript. All authors read and approved the final manuscript.

## Conflict of Interest Statement

No conflict of interest exists in the submission of this manuscript, and the manuscript is approved by all authors for publication.

## Funding Information

This study was funded by the National Key Research and Development Program of China (Nos. 2022YFC2503100 and 2022YFC2503104), the Science and Technology Department of Sichuan Province (No. 2024NSFSC1813), and the Postdoctoral Research Fund of West China Hospital, Sichuan University (No. 2024HXBH020), and the Postdoctoral Fellowship Program (No. CPSFGZC20231825).

## Ethics Statement

The Ethics Committee of West China Hospital of Sichuan University approved the study. Written informed consent was deemed unnecessary by the hospital's institutional review board.
